# Implant survival after graftless sinus floor augmentation in highly atrophic maxillae: a randomized controlled trial in a split mouth study

**DOI:** 10.1186/s40729-021-00387-y

**Published:** 2021-10-18

**Authors:** Suen A. N. Lie, Carine A. W. Leung, Rick M. M. A. Claessen, Hans-Albert Merten, Peter A. W. H. Kessler

**Affiliations:** 1 Department of Cranio-Maxillofacial Surgery, (Head: Prof. Dr. P.A.W.H. Kessler), Maastricht University Medical Centre, P. Debyelaan 25, 6229 HX Maastricht, the Netherlands; 2 GROW School for Oncology and Developmental Biology, Maastricht University Medical Centre, P. Debyelaan 25, 6229 HX Maastricht, the Netherlands; 3 Department of Orthodontics Hannover Medical School, Carl-Neuberg-Straße 1, 30625 Hannover, Germany

**Keywords:** Sinus floor augmentation, Guided bone regeneration, De novo bone formation, Maxillary sinus, Implant survival

## Abstract

**Purpose:**

The success rate of dental implants after graftless sinus augmentation versus conventional sinus augmentation surgery in atrophic maxillae in edentulous patients was investigated.

**Methods:**

This randomized study was performed in ten edentulous patients with marked maxillary atrophy. On the graftless side, the sinus membrane was lifted by a resorbable membrane. The control side was augmented with a mixture of autografts and xenografts. Implant placement followed 6 months postoperatively. Outcomes were implant survival, success of prosthetic rehabilitation and stability of vertical bone gain.

**Results:**

Ten patients were included. Postoperative radiology showed sufficient bone gain on both maxillary sides. Follow-up varied from 57 to 88 months. The conventional side showed significant (*p* = 0.041) more bone gain than the experimental side (respectively, 9.69 mm and 6.20 mm). A total of 59 implants were placed: 30 after conventional, 29 after graftless augmentation. One implant was lost on the conventional side and four on the experimental side. The implant survival was significantly higher on the conventional side (96.7% vs. 86.2%, *p* < 0.001, RR = 4.14). Prosthetic restoration was functionally successful in all cases.

**Conclusion:**

Bone gain and implant survival were significantly lower in the non-grafted side versus the grafted side. Prosthetic rehabilitation was possible in all ten patients. The non-grafted technique may have some potential for clinical use, although it showed poorer results.

*Trial registration* The Netherlands Trialregister. NTR NL3541 (NTR3696). Registered 20 January 2013, https://www.trialregister.nl/trial/3541.

## Background

Extreme atrophy of the edentulous maxilla is a common problem that requires augmentation surgery for achieving a sufficient alveolar bone volume allowing for dental implant placement [1–3]. Lateral sinus membrane elevation for the posterior maxilla [4, 5] is a reliable procedure for augmentation of the maxilla with autogenous, xenogenous or other bone replacement material [6–10].

The sinus lift procedure is not straight-forward and is associated with complications that may compromise the stability of the graft and the overall success of the treatment, such as the perforation of the sinus membrane, which occurs in more than 20% of cases [11, 12]. The use of autogenous bone comes along with donor site morbidity [13–15]. The choice of augmentation technique and augmentation material remains controversial and depends mainly on the degree of atrophy of the upper jaw, the experience of the surgeon, the medical condition of the patient and the medical–technical possibilities of the operating room [3, 5, 16, 17]. The practitioner can solve these problems with circumscribed augmentative surgery using bone graft substitutes, computer-assisted cone beam computed tomography (CBCT)-based computer-guided implant placement and short dental implants [18, 19]. Simultaneous augmentation and implantation in the posterior maxilla is another possibility and has been discussed widely in literature [20–22]. It is acceptable only if the remaining vertical bone height and width are sufficient for primary implant stability.

Ellegaard et al. [23] introduced a sinus lift technique in the posterior maxilla with sinus membrane elevation and simultaneous implant placement without grafting material. Later, Lundgren and co-workers [24] published extensively on this topic and established it in daily practice. Over the years, this technique has been widely used [25–27]. In 2015 we published a variant of this technique, in which we elevated the sinus membrane without augmentation material in severely atrophied edentulous patients in order to place dental implants in a second step. The aim of the study at that time was to investigate spontaneous bone regeneration [28]. The aim of the present study was to evaluate implant survival, success of prosthetic rehabilitation and stability of vertical bone gain on the non-grafted maxillary side compared to conventional sinus floor augmentation with a mixture of autogenous and xenogenous bone.

## Methods

**Fig. 1:**
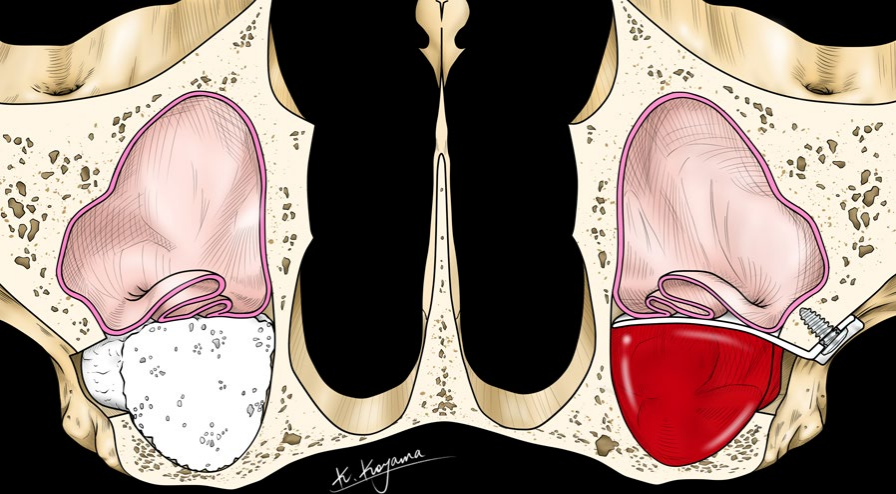
Split mouth study design to compare two different techniques for augmentation of the atrophic posterior maxilla: one side is augmented with a mixture of autogenous and xenogenous bone, on the contralateral side the sinus membrane is lifted and the space is filled with a blood clot

In January 2013, we started our pilot study as a prospective randomized controlled clinical trial in a split mouth study design to compare the efficacy of two different techniques for the augmentation of atrophic posterior maxillae in completely edentulous patients (Fig. 1). This human study design is a translational study that has emerged from a number of animal studies in the past. The reason we developed this particular technique is that the research group already had years of experience and evidence of new bone formation after periosteal elevation with mesh space holders in animals [29–32]. This study [28] was approved by the medical ethics committee of the Maastricht University Medical Center: azM/UM: NL41286.068.12/METC 12-2-066. The study was conducted in full accordance with the principles outlined in the Declaration of Helsinki. All included patients were fully informed about the study design and alternatives and signed the informed consent form.

Ten completely edentulous patients from the outpatient clinic of the Department of Cranio- and Maxillofacial Surgery of the Maastricht University Medical Center (MUMC), with bilateral atrophic maxillae and insufficient total prostheses were included in the study. The inclusion criteria were: complete edentulous, age 18–75 years old, residual bone height of 1–8 mm, bone width of at least 5 mm. Patients with contraindications for general anesthesia, a history of radiotherapy in the head/neck region, treatment with bisphosphonates, poor oral hygiene, uncontrolled diabetes, pregnancy, infection and increased tendency to hemorrhages, were excluded from participation of this study.

The maxillary side being treated with the experimental technique was assigned randomly; a randomization was made in SPSS (statistical package for the social sciences, IBM Corp., Armonk, NY, USA) from patient one to ten. Five papers said “experimental side LEFT” and five said “experimental side RIGHT”, they were placed in ten sealed opaque envelopes by a medical student, without informing anyone about the content. The envelope was opened by the responsible surgeon (SL) on the day of surgery right before the start of the operation. The patients were blinded regarding which side would be the test or control side.

### Surgical procedure

**Fig. 2:**
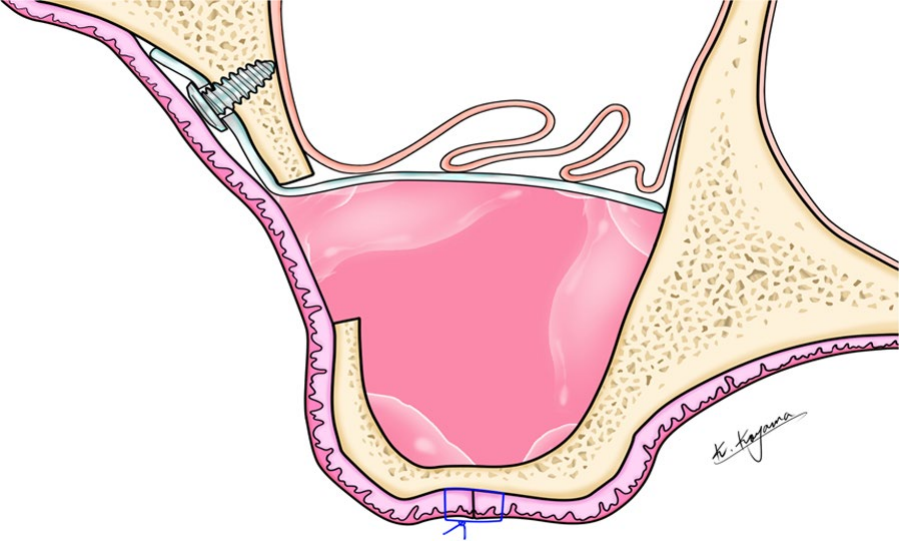
Schematic drawing of sinus membrane elevation stabilized with a resorbable membrane

The operations took place in general anesthesia at the MUMC. All patients were operated by the same surgeon (SL). All patients received 2200 mg amoxicillin/clavulanic acid peri-operatively; this was continued for 7 days postoperatively (Augmentin 500/125 mg, GSK, Brentford, Middlesex, UK, 3×/day). The sinus floor was approached by the lateral window technique. One side was augmented with an equal mix of autogenous bone (anterior iliac crest) and xenogenous bone (Bio-Oss™, Geistlich, Wolhusen, Switzerland). On the randomized test side the maxillary sinus membrane was lifted and stabilized with a resorbable perforated membrane, no bone substitute materials were added (Fig. 2).

The resorbable membrane we used was made of poly(D,L)-lactide (PDLLA) (Resorb X™, KLS Martin, Tuttlingen, Germany). The membrane (40 × 40 × 0.2 mm) was cut to the right size and extra perforations were made. This was followed by heating the membrane in a warm water bath allowing the membrane to be formed to the ideal shape. When the shaped membrane was cooled down, the membrane was rigid and it could create a stable support to keep the sinus membrane elevated. Two resorbable pins (5 mm length) of the Sonic Weld™ system (Sonic Weld™, KLS Martin, Tuttlingen, Germany) were used to fixate the membrane to the lateral wall of the sinus. The distance from the sinus floor to the position of the PDLLA membrane was always determined by the surgeon depending on the anatomical situation and was similar or even higher than on the conventional side. This distance could not be technically measured correctly, but always had to be at least 10 mm in order to be well prepared for the subsequent implant placement of at least 8 mm length. The space created in the sinus was filled with autogenous venous blood. Finally, the wound was closed tightly with resorbable sutures (Vicryl™ Rapid 4 × 0, Ethicon, Johnson & Johnson, New Brunswick, NJ, USA) [28].

According to the study protocol six Straumann® Bone Level implants, three per side, were placed 6 months after the sinus floor augmentation (Straumann AG, Basel, Switzerland). The ideal implant position was planned preoperatively with the aid of the computer and transferred to the operating room using a drilling template (Blue Sky Plan™, Libertyville, IL, USA). Prior to implantation, bone biopsies of 8 mm length were taken exactly in the region of the planned implant placement using a trephine drill. In detail: instead of using a solid twist drill, we used a trephine drill of 2 mm inner diameter and 3 mm outer diameter for the preparation of the implant bed. Cylindrical bone samples from the center region of the augmented maxilla could be obtained for histological evaluation. The histological outcome has been described in detail in an earlier publication [33]. The implantation sites were closed with tensionless continuous sutures (Vicryl™5 × 0).

**Fig. 3:**
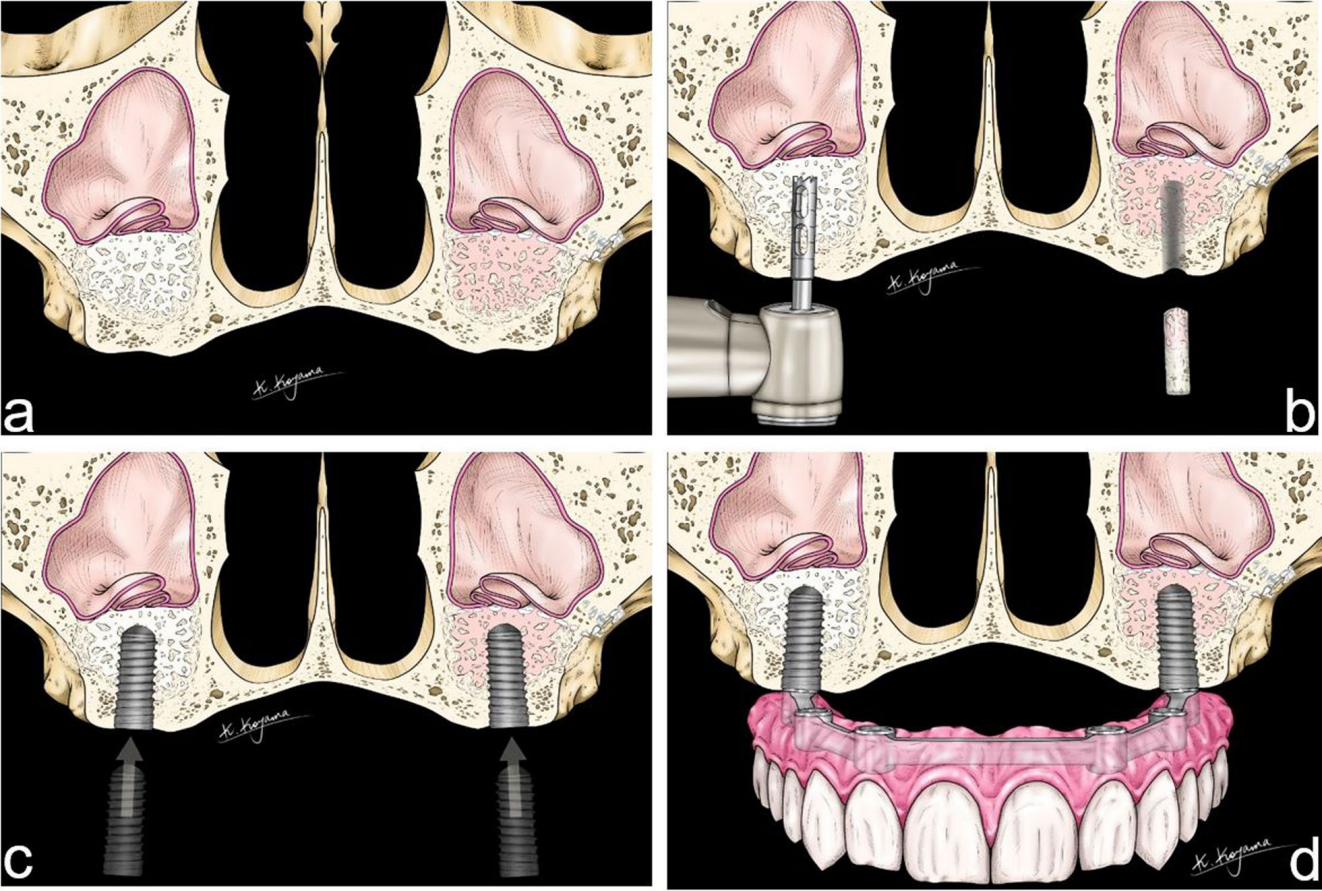
Schematic drawing of implant placement. **a** After 6 months sufficient bone gain was apparent. **b** Bone biopsies are taken with a trephine drill. **c** Implants are inserted at the same place where the bone biopsies were taken: three on each side. **d** After 6 months, the implants are exposed and prosthetic rehabilitation is established

Another 6 months later the implants were exposed, followed by prosthetic rehabilitation by the prosthodontist (Fig. 3). Implant survival and success of prosthetic rehabilitation were documented.

### Radiological follow-up

Radiological follow-up with CBCT (iCatVision™ model 17–19, Imaging Sciences International, Hatfield, PA, USA) was applied preoperatively to the maxillary sinus lift procedure, immediately postoperative and 4 months later.

**Fig. 4:**
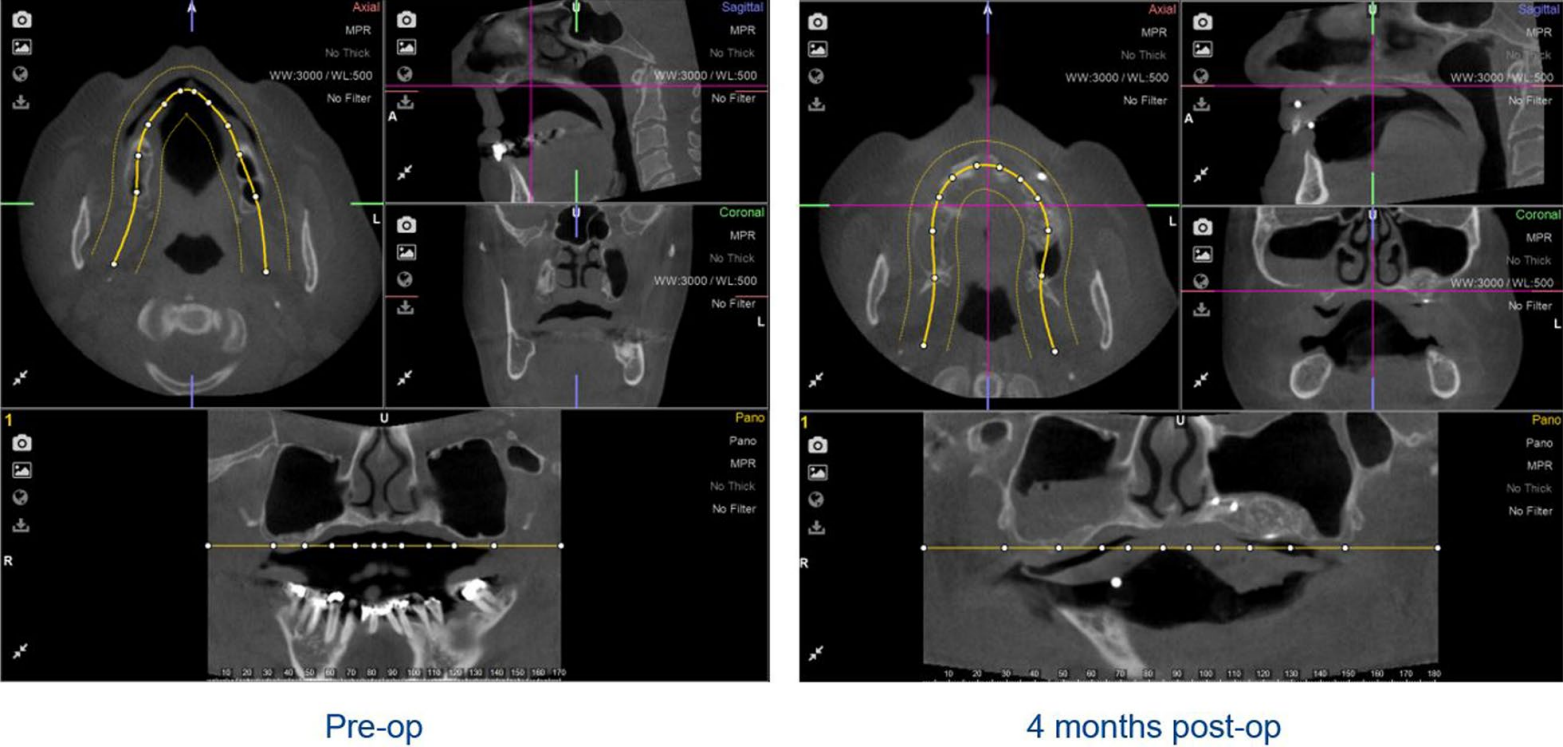
Planes of the preoperative and 4-month postoperative CBCTs in the right orientation: transversal, sagittal and frontal. The lower parts show the defined panoramic plane in which the measurements of bone height were made

The residual vertical bone height from the preoperative CBCT and the vertical bone gain 4 months after sinus membrane elevation were measured on the radiographic images by two independent researchers (SL and CL) and the average was taken. A protocol for these measurements was made: first the CBCT was uploaded in the 3D-planning program (Nemotec™ 3D Scan Dicom, Madrid, Spain), second the CBCT was positioned in a way that the palate was oriented horizontally in the coronal and sagittal planes and the maxilla was placed in the midline in the transversal plane. The third step was to define the panoramic plane of the maxilla, in this plane the bone heights were measured (Fig. 4).

**Fig. 5:**
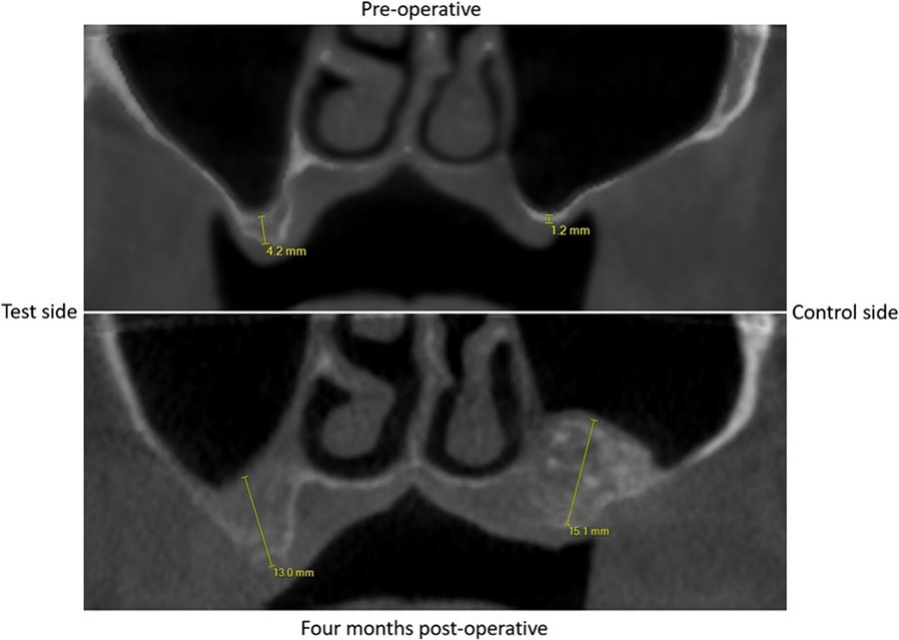
Vertical bone height measurements on two corresponding locations preoperatively and 4 months after augmentation

In the augmented posterior region of the maxilla, two locations were marked on each side where vertical height measurements were taken. As described previously, the CBCT, 4 months post-augmentation, was used to produce a drilling guide. The sites where the two posterior implants were placed, pre-planned with the implant 3D-planning software, were exactly the sites where bone height was measured. First the bone height in the 4-month postoperative CBCT scan was documented, and afterwards the bone height at exactly this point in the preoperative scan was identified (Fig. 5).

All trephine biopsies have showed us new bone formation in macro- and microscopy [33] on both sides, with less bone density on the experimental side. This explains a lower radiographic opacity on the experimental side. Therefore, we made the sensible decision to consider any radio-opacity from gray to white as bone.

### Implant survival

Six months after implant placement, the implants were exposed. Implant survival was assessed radiologically at the day of exposure. One year after functional loading of the upper prosthesis another radiological check was performed and implant survival was registered again. The clinical follow-up after implant placement varied from 57 to 88 months.

The criteria of Albrektsson et al. [34] were used to evaluate the success of the implants:

- No signs of radiographic peri-implant translucency,
- Vertical bone resorption less than 0.2 mm per year after the first year under loading,
- The clinical appearance must be free of pain, infections, neuropathies.

### Success of prosthetic rehabilitation

Prosthetic constructions were described as fully functional if they did not show any vertical positional change, had a perfect fit and did not show any occlusal disturbances.

### Statistical analysis

Descriptive statistics were performed using SPSS™ (statistical package for the social sciences version 25, IBM Corp., Armonk, NY, USA). Mean and standard deviations (SD) were given. The mean difference and *p*-value was determined by a one-sample t-test.

## Results

A total of ten patients, four males and six females, were treated according to the study protocol. The age ranged from 50 to 70 years with an average of 60.5 years (SD 7.7). A bilateral sinus membrane elevation had been performed in all patients in a split mouth design. Six months after maxillary sinus augmentation, only one patient showed too little vertical bone gain on the graftless side in the preoperatively planned implant site. On this side, only two implants could be placed. A total of 29 implants were inserted, 14 in the test group and 15 in the control group. During the sinus lift procedure two small membrane lacerations of about 1 mm occurred at the test side and two sinus membrane perforations (1 and 2 mm in length) occurred at the control side. These lacerations did not need any treatment. In one patient, the incision site for bone harvesting became infected one week postoperatively. The patient was treated with antibiotics (Augmentin 500/125 mg, GSK, Brentford, Middlesex, UK) for 7 days. The wound healed within 3 weeks. No other postoperative complications occurred. No patients dropped out of the follow-up. The follow-up period ranged from 4 years and 9 months to 7 years and 4 months.

### Radiology

Six months after augmentation all patients showed sufficient new bone formation, which allowed for dental implant placement. The non-augmented and the augmented sides were compared and both showed sufficient new bone formation. However, the non-augmented side showed less opacity suggesting less bone density or less presence of mature bone (see Figs. 4 and 5).

The two assessors (SL and CL) followed the protocol to measure bone height gain separately, so that the results would be as accurate and reliable as possible. An inter-observer variability analysis was performed which showed a rate of 0.98, from which we can trust that the numbers in the results are reliable. The gain in vertical bone height is documented in Table 1.

**Table 1:** Radiologically assessed vertical bone height gain 4 months after sinus membrane elevation/augmentation

10 patients, CBCT		Bone height					
		Experimental side (mm)			Conventional side (mm)		
		Before	After	Gain	Before	After	Gain
1	Ant	3.73	8.92	5.19	4.57	16.00	11.43
	Post	7.28	7.35	0.07	7.54	18.64	11.10
2	Ant	1.26	16.64	15.38	8.11	13.11	5.00
	Post	2.77	21.05	18.28	0.83	13.68	12.85
3	Ant	1.69	10.18	8.49	3.12	13.63	10.51
	Post	1.40	8.93	7.53	8.23	11.16	2.93
4	Ant	5.55	8.50	2.95	0.88	11.73	10.85
	Post	5.26	8.12	2.86	4.57	8.86	4.29
5	Ant	6.78	12.75	5.97	2.79	14.44	11.65
	Post	4.60	8.21	3.61	6.61	12.20	5.59
6	Ant	6.60	10.25	3.65	6.08	13.54	7.46
	Post	6.86	11.02	4.16	5.01	12.62	7.61
7	Ant	3.35	12.38	9.03	5.36	8.02	2.66
	Post	7.23	11.52	4.29	6.96	9.20	2.24
8	Ant	0.99	10.23	9.24	3.25	15.99	12.74
	Post	6.84	7.65	0.81	2.59	20.56	17.97
9	Ant	11.69	11.73	0.04	4.23	16.79	12.56
	Post	6.44	9.35	2.91	4.01	20.08	16.07
10	Ant	1.97	12.42	10.45	3.69	14.03	10.34
	Post	0.98	10.48	9.50	2.12	14.26	12.14
Mean (sd)	Ant	4.36 (3.35)		7.20 (4.29)	4.21 (1.99)		9.88 (3.76)
	Post	4.97 (2.44)		5.19 (5.52)	4.85 (2.49)		9.49 (5.55)
	Overall	4.66 (2.87)		6.20 (4.92)	4.53 (2.22)		9.69 (4.62)

The average residual bone of the test side was 4.66 mm (SD 2.87 mm) and of the conventional side 4.53 mm (SD 2.22 mm). The paired samples t-test showed no significant difference between the test and control sides (*P* = 0.866).

In the test group vertical bone gain ranged from 0.04 mm to 18.28 mm with an overall mean of 6.20 mm (SD = 4.92 mm). The control group showed a range in vertical bone gain from 2.24 mm to 17.97 mm with an overall mean of 9.69 mm (SD = 4.62 mm). The one-sample t-test showed a mean difference between the test and control side of −3.49 mm (95% CI −6.82 to −0.16). The vertical bone gain height on the experimental side was significantly lower than on the contralateral control side (*P* = 0.041).

### Implant survival and prosthetic survival

Implants of appropriate width and length (Bone Level Implants, Straumann®, Basel, Switzerland) were placed in the maxilla (Table 2).

**Table 2:** Characteristics of the inserted bone-level implants

Implant sizes (diameter × length in mm)	Test side			Control side		
	Anterior	Mid	Posterior	Anterior	Mid	Posterior
1	3.3 × 8	3.3 × 8	3.3 × 8	3.3 × 10	4.1 × 12	4.1 × 12
2	3.3 × 10	4.1 × 10	4.1 × 12	3.3 × 10	3.3 × 12	4.1 × 12
3	3.3 × 12	4.1 × 10	3.3 × 10	3.3 × 10	4.1 × 12	4.1 × 12
4	3.3 × 8	3.3 × 8	3.3 × 8	3.3 × 10	3.3 × 10	3.3 × 8
5	4.1 × 10	4.1 × 12	3.3 × 8	4.1 × 10	4.1 × 12	4.1 × 10
6	4.1 × 10	3.3 × 10	3.3 × 10	4.1 × 10	4.1 × 10	3.3 × 10
7	3.3 × 10	4.1 × 10	4.1 × 10	4.1 × 10	4.1 × 10	4.1 × 10
8	4.1 × 10	4.1 × 10	3.3 × 8	3.3 × 10	4.1 × 10	4.1 × 10
9	3.3 × 12	3.3 × 12	4.1 × 10	4.1 × 10	4.1 × 12	4.1 × 10
10	4.1 × 10	4.1 × 12	–	4.1 × 10	4.1 × 12	4.1 × 10

Implant failure was seen in three patients. During the second stage surgery and uncovering of the fixtures in each of two patients one implant on the test side was lost. A third implant was lost in another patient on the control side. In this particular patient another implant on the test side was lost 3 months after functional loading. Again, another patient who had lost an implant during exposure on the test site lost another implant on the test site 8 months after functional loading. During the last follow-up (varying from 57 to 88 months), no more implants were lost (Table 3).

**Table 3:** Implant failure

10 patients	Test side	Control side
Total implants placed	29	30
Implant failure during exposure	2 (middle, posterior)	1 (anterior)
Total implant failure one year after functional loading	4 (3 middle, 1 posterior)	1
Total implant failure at last follow-up	4	1
Overall implant survival (%)	86.2	96.7

In summary, a total of five implants were lost during follow-up: four at the test side and one at the control side. The overall implant survival was 86.2% on the test and 96.7% on the control side (*P* < 0.001). The risk ratio of losing an implant on the test side is 4.14 (95% CI 1.88–6.39).

If anteriorly placed implants are excluded, implant survival is 78.9% on the test side and 100% on the control side (*P* = 0.125).

The remaining implants fulfilled the criteria of implant success as defined by Albrektsson in [34].

Table 4 shows the data on the existing residual bone before implant placement at the exact sites where the implants were later lost. The table shows that the four lost implants on the test side had less residual bone than the average. Three locations even deviated by more than one standard deviation.

**Table 4:** Residual bone height before sinus floor augmentation on the sites where implants were later lost

Implant loss	Side	Location	Residual bone (mm)
Patient 1	Conventional	Anterior	6.41
	Experimental	Middle	1.26
Patient 2	Experimental	Middle	1.69
	Experimental	Posterior	1.40
Patient 3	Experimental	Middle	3.35

**Fig. 6:**
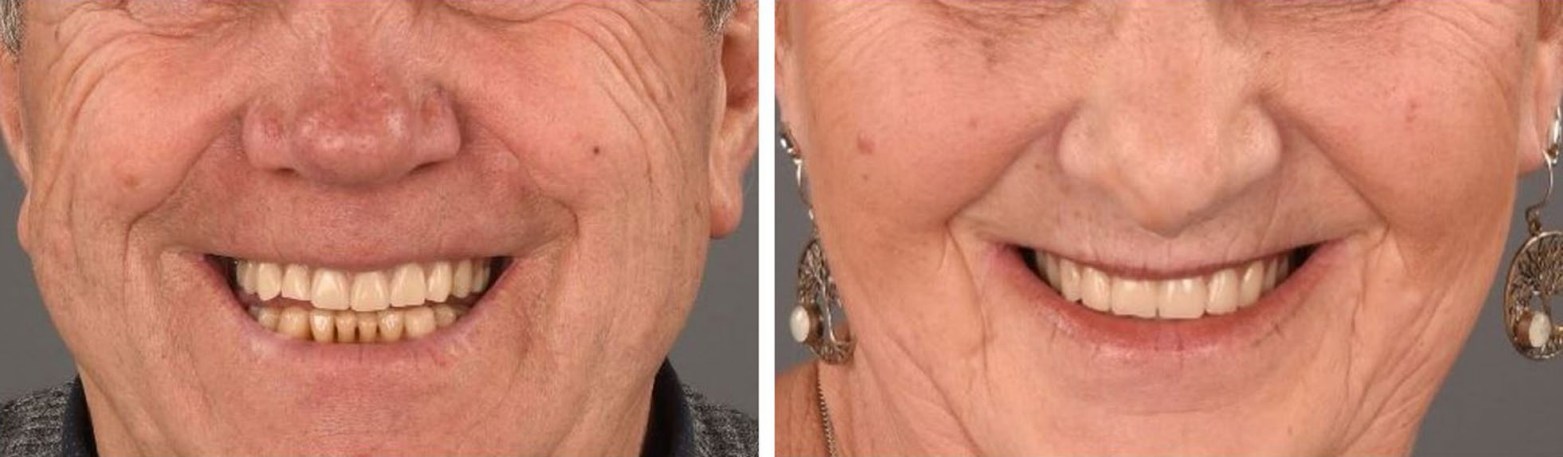
Esthetic smile results of the upper denture

**Fig. 7:**
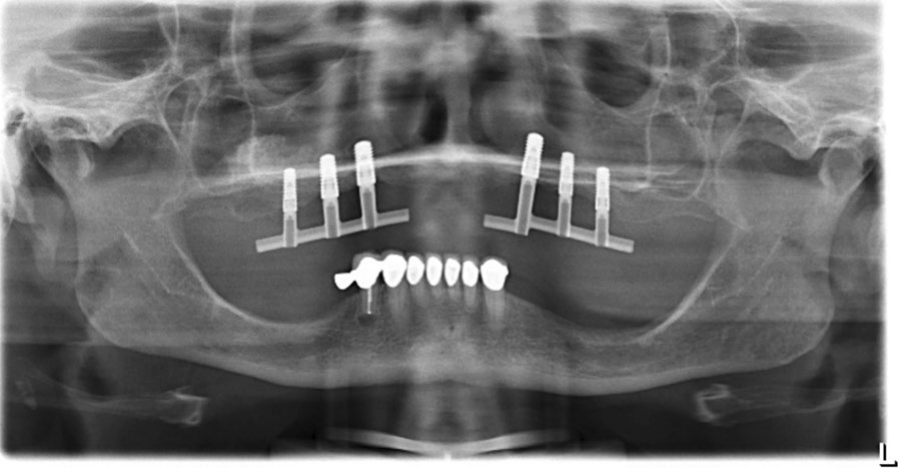
Panoramic X-ray of a bar-retained superstructure on six implants in the augmented maxilla (test: left side, control: right side)

All patients received a prosthetic restoration (Figs. 6 and 7) on a bar-retained superstructure. Despite the loss of implants, all prosthetic restorations were functional until the end of follow-up according to the definition presented above.

## Discussion

At the end of this study, we wondered whether graftless sinus floor augmentation, as we investigated it, could be a real alternative to the many existing techniques described in the literature to create a stable bone supply in the atrophic posterior maxilla pre-implantologically. Our group recently published a systematic review [27] that concluded that both non-grafted maxillary sinus lifts and conventional sinus lifts with augmentation materials have a high implant survival rate (97.92% and 98.73%, respectively). Aghaloo et al. have addressed this question in 2007 and again in 2016 [35, 36]. The article from 2016 is a systematic review article that deals with scientific contributions from 1980 to 2014 and describes the five most important augmentation procedures. All five techniques—guided bone regeneration, interpositional grafting, maxillary sinus augmentation, inlay and onlay grafting or combinations of the above can be successfully applied in pre-prosthetic augmentation surgery of the maxilla. The technique of sinus floor grafting without graft material has not been studied, as this technique was quite new. However, there is a consensus in the relevant literature that if the residual height of the alveolar process in the posterior maxilla is less than 6 mm, augmentation measures are usually recommended [2].

Established and successful grafting procedures for augmentation on the posterior edentulous atrophic maxilla exist, which offer long-term implant survival of 85 to 100% [37–39]. Nkenke et al. in 2004 and Kessler et al. in 2005 conducted studies on large patient series in which autogenous bone was harvested from the anterior or posterior iliac crest in pre-prosthetic indications [13, 40]. If large bone volumes are required for three-dimensional maxillary augmentation with lateral maxillary widening, these techniques can still have their justification. In 2014, Nkenke and Neukam published an article dealing only with comparative clinical trials on the harvest of autogenous bone grafts [41]. Six intraoral or distant donor sites were identified. From the patients' perspective, intraoral bone harvesting at the ramus was preferred. Interestingly, the comparison of chin bone harvesting with iliac crest bone harvesting led to a preference for pelvic bone harvesting, although this requires treatment under general anesthesia. This choice was justified by the high morbidity of chin bone harvesting. Pain, loss of superficial skin sensitivity and wound healing disorders led to a high morbidity rate after chin bone harvesting. This is astonishing, since the literature often suggests that intraoral bone harvesting causes less problems than harvesting at the iliac crest. There is sufficient literature on this issue that we cannot go into detail here. Nevertheless, the unavoidable morbidity of autogenous bone harvesting was the main motivation to submit our study to the Ethics Committee in the first place, which considered the argument of non-existent morbidity in our study protocol as decisive for approval. However, it should not be forgotten that only autogenous bone grafts provide all biological advantages desired at a defect side, such as: scaffold for osteoconduction, growth factors for osteoinduction and progenitor cells for osteogenesis, which altogether seems to be decisive with regard to bone volume stability and the structure of augmented bone sites [42]. With autogenous bone as bone source for augmentation exceptionally high implant survival of up to 100% after cumulative 3-year analysis after placement of implants in the maxilla regardless of source, chin or iliac crest bone were reported in literature [43].

The use of bone replacement materials is the classical alternative to autogenous bone. An almost unmanageable amount of alternative bone product is offered. Bone substitutes can be used just as successfully as autogenous bone. Al-Nawas and Schiegnitz published a systematic review and meta-analysis on bone substitutes as augmentation materials in maxillary sinus floor augmentation. The mean implant survival rate was 98.6% for implants placed in sinuses augmented with bone substitute materials. In the comparison groups, sinus floor augmentations with a mixture of autogenous bone and bone replacement material and autogenous bone were used exclusively. In the former group the mean implant survival was 88.6%, in the latter 97.4%. All procedures appear to allow long-term implant survival [44]. Similar results were published by many other authors [1, 45, 46].

Even the question whether sinus lifting is necessary at all is difficult to answer, if short implants of less than 8 mm can successfully be loaded in maxillary bone with a residual height of 4 to 6 mm [47]. However, their long-term prognosis is still unknown. This statement must be critically evaluated and can only be seen in the interaction of augmentation, implantation and prosthetic rehabilitation. In most patients, there is atrophy of the maxilla as well as atrophy of the edentulous mandible. If the intermaxillary space becomes too large as a result, short implants may fail if the ratio of the length of the implant and the height of the prosthetic structure is also unfavorable to the implant length. In a recently published study, Al-Nawas and Schiegnitz address the question whether narrow-diameter implants (mini implants) in the atrophic jaw are successful. They warn against mini-implants in the masticatory posterior region [48]. The same could also apply to short implants with unfavorably high prosthetic abutments. In addition, dental implant manufacturers often only guarantee the success of their products if the implant–prosthetic construction aspect ratio is 1:1 [49].

In this context, Summers' technique, which allows the insertion of longer implants, should not go unmentioned [50]. One advantage of the crestal sinus lift, a "minimally invasive" technique, is that it is less traumatic for the patient than the external sinus lift. The narrow and complex surgical access via the burr hole of the crestal sinus lift requires a high degree of skill on the part of the surgeon. An equally important factor of the crestal sinus lift according to Summers is the augmentation height of about 4 to 5 mm that is possible [51].

Ellegaard et al. [23] and later Lundgren [24] were the first to describe successful implantation after sinus membrane elevation without the use of any bone grafts or applying the crestal sinus lift. A coagulum is formed after creating a bone wound by removing the sinus membrane from the floor of the sinus, the lateral and medial sinus walls. The osteogenetic capacity of the surrounding bony walls on at least three sides of the sinus leads to the formation of bone-forming callus from the coagulum. The smaller this space is, the more stable is the coagulum formed in this space. When the space is bigger, like in completely edentulous maxillae, this may lead to a less stable callus. When no bone grafts are used in a sinus membrane elevation, this space does not contain a scaffold [33].

This study is the first study about the implant survival after graftless maxillary sinus membrane elevation that included patients that were all completely edentulous. The use of a resorbable membrane from material like PDLLA has the advantage that no material had to be removed in a second surgery and the crucial region of interest would stay untouched when the implants were placed. The insertion of the implants was challenging because the usual feeling of insertion resistance was missing. As indicated above in the preimplantation radiographic analysis, bone maturation was not yet complete at implant placement or was completed without sufficient calcification. Next to this, we do not exactly know what the resorbable material would do and what influence it has on bone growth. It is known that it is not always fully absorbed after sinus membrane elevation. This was closely studied in a publication of Cricchio et al. [52, 53]. To discover what the PDLLA membrane did in our human study, we should have retrieved histological evidence including the sinus membrane. Since this would bring too much damage to the patient, it would not be approved by the medical ethics committee.

A limitation of the technique used in this study is that the open window to the sinus cannot prevent the growth of granulation tissue into the defect. On the other hand, the periosteum directly covers the opening to the maxillary sinus. There is a possibility that the bone-forming capabilities of the periosteum play a beneficial role in stimulating bone growth.

We trusted the functional biological process that functional loading will result in bone apposition and that the PDLLA membrane would not interfere at implant level. Nevertheless, we can conclude from these clinical results that the implants are osseointegrated. Only long-term (> 10 years) follow-up might show, if the functional loading due to the placement of dental implants in conjunction with appropriate prosthetic rehabilitation will render a stable callus in a non-grafted atrophic maxillary sinus. Further studies have to prove, if earlier or maybe later implant placement will render better results with regard to implant survival. The staged procedure we chose was based on the empirically gained experience of conventional implantology.

This study has some limitations: the sample size is small, there was no option to take bone biopsies with inclusion of the sinus membrane or retrieve implants for histological prove of implant osseointegration. In addition, we know that the posterior maxilla is often more atrophied than the anterior. If we exclude the anteriorly placed implants, we see a survival rate of 100% on the conventionally treated side versus 78.9% on the side treated without bone graft (not significant).

We can also discuss whether the higher implant loss on the test side could be caused by the very small amount of residual bone shown in Table 4. In a next study, it might be interesting to exclude patients with less than 4 mm of residual bone. In this way, we could investigate whether the experimental technique leads to better implant survival.

Furthermore, a uniform definition of the term "loss of function" after prosthetic restoration does not exist and whether there will be one in the near future remains questionable. Often it remains unconsidered or undefined on the basis of which criteria the end of the functional phase of a dental restoration is reached. Mostly retrospective case series with at least 100 patients and an observation period of at least 3 years allowed a study-related estimation of the survival of prosthetically restored implants (implants as surrogate parameters), but the different endpoints as well as the study design (case series) or different forms of prosthetic rehabilitation do not allow pooling of the results [54–57]. The 5-year implant success rates varied between 69.5% and 98.4% (the corresponding 10-year success rates between 79.4% and 94.3%), depending on the prosthesis used [58]. The long time periods make it clear that the event "loss of function" usually occurs only after a very long time, if one has a normal clinical use period in mind. Such a long time can often hardly be waited for results in randomized trials. So far, the results of implant survival in this study show acceptable results since all prosthetic restorations were still functioning.

## Conclusions

We conducted an experiment with completely edentulous humans with atrophic maxillae by comparing conventional augmentation procedures with a sinus membrane lifting technique without using any bone grafts. The results showed new bone formation on both test sides. However, the non-grafted side showed significant less bone gain than the grafted side, with an overall mean of, respectively, 6.20 mm (SD 4.92) and 9.69 mm (SD 4.62).

Implant survival was 86.2% in the non-grafted side vs. 96.7% in the grafted side (*P* < 0.001). Prosthetic rehabilitation was possible in all ten patients with reasonable patient satisfaction. At the last follow-up all prostheses were functional.

Within the limitations of this study, we can conclude that the non-grafted technique might have some potential for clinical use, although it showed a higher implant failure. This technique in the complete edentulous maxilla is a challenging procedure. It can be applied to avoid the use of any bone grafts and reduce morbidity in patients where this is favorable. Further research with more statistical power will be necessary to support the hypothesis of this study.

## Data Availability

The datasets used and/or analyzed during the current study are available from the corresponding author on reasonable request.

## Abbreviations

CBCT: Cone beam computed tomography
MUMC: Maastricht University Medical Center
PDLLA: Resorbable membrane we used was made of poly(D,L)-lactide
SD: Standard deviation
